# Health on the Web: Randomised Controlled Trial of Online Screening and Brief Alcohol Intervention Delivered in a Workplace Setting

**DOI:** 10.1371/journal.pone.0112553

**Published:** 2014-11-19

**Authors:** Zarnie Khadjesari, Nick Freemantle, Stuart Linke, Rachael Hunter, Elizabeth Murray

**Affiliations:** 1 Research Department of Primary Care and Population Health, University College London, Royal Free Hospital, London, United Kingdom; 2 Camden and Islington NHS Foundation Trust, London, United Kingdom; University of Newcastle, Australia, Australia

## Abstract

**Background:**

Alcohol misuse in England costs around £7.3 billion (US$12.2 billion) annually from lost productivity and absenteeism. Delivering brief alcohol interventions to employees as part of a health check may be acceptable, particularly with online delivery which can provide privacy for this stigmatised behaviour. Research to support this approach is limited and methodologically weak. The aim was to determine the effectiveness of online screening and personalised feedback on alcohol consumption, delivered in a workplace as part of a health check.

**Methods and Findings:**

This two-group online individually randomised controlled trial recruited employees from a UK-based private sector organisation (approx. 100,000 employees). 3,375 employees completed the online health check in the three week recruitment period. Of these, 1,330 (39%) scored five or more on the AUDIT-C (indicating alcohol misuse) and were randomised to receive personalised feedback on their alcohol intake, alongside feedback on other health behaviours (n = 659), or to receive feedback on all health behaviours except alcohol intake (n = 671). Participants were mostly male (75%), with a median age of 48 years and half were in managerial positions (55%). Median Body Mass Index was 26, 12% were smokers, median time undertaking moderate/vigorous physical activity a week was 173 minutes and median fruit and vegetable consumption was three portions a day. Eighty percent (n = 1,066) of participants completed follow-up questionnaires at three months. An intention to treat analysis found no difference between experimental groups for past week drinking (primary outcome) (5.6% increase associated with the intervention (95% CI −4.7% to 16.9%; p = .30)), AUDIT (measure of alcohol-related harm) and health utility (EQ-5D).

**Conclusions:**

There was no evidence to support the use of personalised feedback within an online health check for reducing alcohol consumption among employees in this organisation. Further research is needed on how to engage a larger proportion of employees in screening.

**Trial Registration:**

International Standard Randomised Controlled Trial Number Register ISRCTN50658915

## Introduction

Alcohol misuse is among the leading risk factors for disease burden across the globe, after high blood pressure and smoking [1]. In England, the prevalence of alcohol intake is higher in working men and women than the unemployed, with consumption rising with earnings [2], and alcohol-related harm costs the workplace around £7.3 bn (US$12.2 billion) a year (2009/2010 prices) through lost productivity and absenteeism [3]. Screening and brief intervention (SBI) is an effective way of reducing hazardous alcohol-intake to safer levels [4], [5], with a number needed to treat of eight [4]. However, barriers to the delivery of SBI in primary care [6]–[11], where the bulk of the evidence is based [4], [5], [12]–[16], prevents widespread dissemination. One way of addressing these barriers, advocated by the National Institute for Health and Care Excellence (NICE) [16], is to investigate the effectiveness of SBI in non-medical settings, such as the workplace, particularly in view of the high costs of alcohol misuse to employers.

There have been relatively few trials evaluating the effectiveness of SBI for alcohol misuse in the workplace setting. In 2009, a systematic review of workplace interventions for alcohol-problems [17] identified seven randomised trials [18]–[24] evaluating brief interventions or counselling-based interventions. Although there was some evidence that brief intervention and psychosocial skills training are effective in this setting, studies were fraught with methodological limitations including lack of exposure to the intervention, contamination of the intervention, and control groups obtaining access to the intervention.

One of the challenges with delivering SBI to employees in the workplace is the stigma associated with accessing services for alcohol misuse in this setting [25]. Electronic screening and brief intervention (eSBI) allows employees to access the intervention in a private and confidential setting. The Internet enables the delivery of personalised feedback, which can be tailored according to baseline data and delivered instantaneously on any device with access to the Internet, hence at low cost and with wide reach and convenience. Some studies have found Internet-based interventions to be effective at reducing alcohol consumption when compared with minimally active comparator groups (e.g. assessment-only), with a small number of studies finding them to be as effective as active comparator groups, such as in-person cognitive behavioural therapy [26]–[28], but most of the evidence is based in student populations [29]–[31].

Another way of addressing the stigma surrounding SBI for alcohol in the workplace may be to deliver it in the context of a health check [25]. In 2009, a large feasibility study found SBI delivered in person by occupational health to be acceptable to employees of a Scottish Local Authority, where 92% of respondents to a general lifestyle survey were reportedly happy to be asked about their drinking [32]. Online health checks have the additional advantage of ensuring that alcohol questions are asked alongside other behaviours and not avoided, which is a concern when brief advice is delivered in-person. A top priority of Public Health England for 2013/14 is to reduce preventable mortality and morbidity associated with alcohol consumption, smoking, poor diet and lack of exercise [33], therefore an online intervention that combines brief advice on all of these health behaviours is ideal for the workplace setting.

The aim of this study was to determine the effectiveness and cost of screening and personalised feedback on alcohol consumption, delivered as part of an online health check in a workplace setting. It was hypothesised that participants receiving the personalised feedback on alcohol consumption would reduce their alcohol intake more than those not receiving the feedback.

## Methods

### Design

This was a two-group, individually randomised controlled trial, conducted entirely online. Ethical approval was granted from University College London (UCL) Research Ethics Committee (4213/001) and the trial was registered with UCL's data protection officer. The trial protocol has been published [34] and preliminary data were published as conference proceedings [35]. The trial registration number is ISRCTN50658915 (www.controlled-trials.com/ISRCTN50658915). The protocol for this trial and supporting CONSORT checklist are available as supporting information; see Checklist S1 and Protocol S1.

### Setting and participants

This study was conducted online in a large UK-based organisation with an international workforce of around 100,000, with the majority of employees based in the UK. The company has an active occupational health team which runs frequent campaigns aimed to increase awareness and understanding of health behaviours, engage people to take personal responsibility for their health, and to change attitudes and behaviours. Campaigns often include online information, assess risk, facilitate monitoring activity, share information, present prizes to winners of competitions, and include: virtual gyms, road shows, health fairs and articles in newsletters. The organisation has worked with other academic institutions, which meant its employees were familiar with the process of taking part in research and answering questions about their health via the workplace. Employees aged 18 years and above were eligible to take part if their drinking put them at increased risk of alcohol-related harm, as indicated by a score of five or more on the Alcohol Use Disorders Identification Test – Consumption (AUDIT-C - three-item screening tool for alcohol misuse) [36], in-line with clinical guidance in England [37].

### Recruitment

Employees were invited to take part in a confidential online health check and receive personalised feedback on a range of behaviours known to impact on their health and wellbeing. The study advertisement was edited by the organisation's occupational health and communications teams into their “language” and posted on the company's web-portal in August 2012 for three weeks. All employees login through this portal to access any resource or service. We had intended to recruit participants to the study via individual email sent from the occupational health team to all employees [34], but this was against company policy. The advertisement invited employees to complete an online health check as part of a study led by researchers from UCL. If interested in learning more about the study, employees clicked on a hyperlink which took them to the study website. The study website provided information on the study procedure and made it clear that the organisation would not know whether individual employees had taken part, and that all the information provided was confidential and will only be seen by the researchers in an unidentifiable format. Study information was followed by an online consent form that asked employees to agree to complete a series of questions now and possibly again in three months' time (where those scoring five or more on the AUDIT-C would be followed up), on behaviours that affect their health. Participants were not aware they were taking part in a trial. Consent was followed by a mandatory request for contact information, including email address, postal address and telephone number.

The online health check asked employees about their height and weight (for calculating Body Mass Index - BMI), alcohol consumption, smoking status, fruit and vegetable consumption and level of physical activity. Respondents were then asked for some basic demographic information (see below for further details), before receiving immediate online tailored feedback which either 1) reinforced healthy behaviour and reminded people of recommended guidelines, or 2) encouraged a change in behaviour by highlighting the risk associated with not meeting the recommended guidelines. Feedback for BMI was categorised as underweight, healthy weight, overweight, obese or morbidly obese. Feedback was accompanied by links to relevant NHS Choices webpages and the organisation's own behaviour specific webpages. Employees scoring less than five on the AUDIT-C received feedback that their drinking was within recommended limits, a reminder of these limits, and feedback on the other health behaviours; they were not eligible for the trial and had no further contact with the study team. Employees scoring five or more on the AUDIT-C were automatically entered into the trial and randomised to the intervention or the control group.

### Intervention

The intervention group received feedback on all health behaviours assessed in the health check, as detailed above, the only difference being their alcohol feedback, which provided criterion or risk-based feedback on the potential harm of drinking above recommended limits [38]. Brief advice is advocated by NICE for people drinking at hazardous and harmful levels [16]. Included in this feedback was information and a hyperlink to an additional web-based resource, Down Your Drink (DYD), which was described as a resource for participants who wanted help to reduce their drinking. DYD is an extended online alcohol intervention based on the principles of motivational interviewing, cognitive behavioural therapy, behavioural self-control, and relapse prevention (www.downyourdrink.org.uk) [39]. Hence the core intervention was screening and personalised feedback, with the option of a more extended intervention for those who wanted.

### Control

The control group received feedback on all health behaviours except alcohol consumption, in a wait-list design. Participants in both arms of the trial received feedback on their alcohol intake after completion of three month follow-up measures. It was not possible to assess long-term differences between groups as both experimental arms received the intervention (instantaneous personalised feedback on alcohol consumption) after three months.

### Data collection

#### Baseline data collection and potential sources of bias

Baseline data were collected in the form of an online health check which asked employees about a range of behaviours, as described above. Alcohol consumption questions were the 3-item AUDIT-C questionnaire [36], which consists of the first three consumption questions of the World Health Organisation's (WHO) Alcohol Use Disorders Identification Test (AUDIT), where scores range from 0 to 12. Clinical guidelines in England advise using a score of five or more to indicate alcohol misuse [37]. Employees were also asked: whether they smoked; the average number of portions of fruit and vegetables they consumed per day, where the recommended number of portions in the UK is 5 or more; the average number of minutes spent undertaking light, moderate and vigorous activity a week, where more than 150 minutes of moderate or vigorous activity is recommended a week in the UK. Participants were then asked to provide demographic information before receiving feedback, this included sex, age, marital status, number of children, ethnicity and occupational classification (as defined by the organisation).

All baseline data were collected prior to randomisation. Respondents scoring five or more on the AUDIT-C were randomised by simple randomisation via computer-generated randomisation software to experimental groups in an automated process, therefore concealing allocation. It was not possible to blind participants to randomised groups as they either received the alcohol feedback or they did not. Participants received automated feedback online without facilitation by a researcher, health professional or member of occupational health; therefore obviating the need for blinding intervention providers. In addition, outcome data were self-completed by the participant eliminating bias introduced by an outcome assessor.

### Outcome measures and follow-up procedure

Participants were contacted by email three months after completing the online health check and asked to complete follow-up questionnaires online via a hyperlink. The primary outcome measure was the TOT-AL, an online measure of self-reported past week alcohol intake that calculates UK units consumed per week (where 1 UK unit  = 8 g of ethanol) [40]. Secondary outcome measures included: 1) the full Alcohol Use Identification Test (AUDIT), the WHO measure of alcohol-related harm [41] with time frame amended to past three months, where scores range from 0 to 40 and a score of eight or more indicates alcohol misuse; 2) health state, measured by the EQ-5D index [42], 3) number of days of sickness absence in the past three months (self-reported); 4) primary and secondary health care resource use in the past three months (self-reported).

Participants received up to three emails at five day intervals, followed by a letter and a phone call. The letter provided the URL to the data collection page, but also contained a paper-based version of past week drinking and the health service utilisation questions. Postal questionnaires displayed the participant's unique identifier and were returned in pre-paid envelopes. Postal questionnaire data were entered into a separate database by an independent researcher and amalgamated into the main database by the trial statistician. Telephone calls served as prompts to complete either online or paper-based measures.

### Sample size

736 participants in each group at 3 month follow up would have provided 90% power with 5% significance to determine a 20% reduction in alcohol consumption. Making a 25% allowance for loss to follow-up required randomising 920 participants to each group (n = 1,840). Extensive steps were taken to minimise loss to follow-up through tailored reminders as described above.

### Data analysis

Analyses compared the primary and secondary outcomes between groups at 3 months, following the intention to treat principle. The principal analysis was conducted using a generalised linear model with identity link and Gaussian error. The baseline AUDIT-C score was included as a patient level explanatory variable, along with a classification variable for workplace and for randomised group. Analogous models were conducted for secondary outcomes.

### Costing

Self-reported use of health care resources and number of sick days were used to calculate the mean cost per participant to the English National Health Service (NHS) and employer. Unit costs for health care resource use were obtained from published sources [43]. The cost to the employer was calculated as the average cost per participant of sick leave. This was calculated as the cost of a sick day is the amount earned by the employee per day, using gross weekly pay published by the Office for National Statistics (ONS) [44]. Costs per day were broken down by occupational classification and gender. All costs reported are for the year 2012 and are in British pounds. Bootstrapping was used to estimate differences in the mean total cost (NHS plus employer costs) between the two groups.

## Results

### Recruitment

A total of 3,375 employees completed the online health check in the three week recruitment period, which constituted around 3% of the organisation's total international workforce.

**Figure 1:**
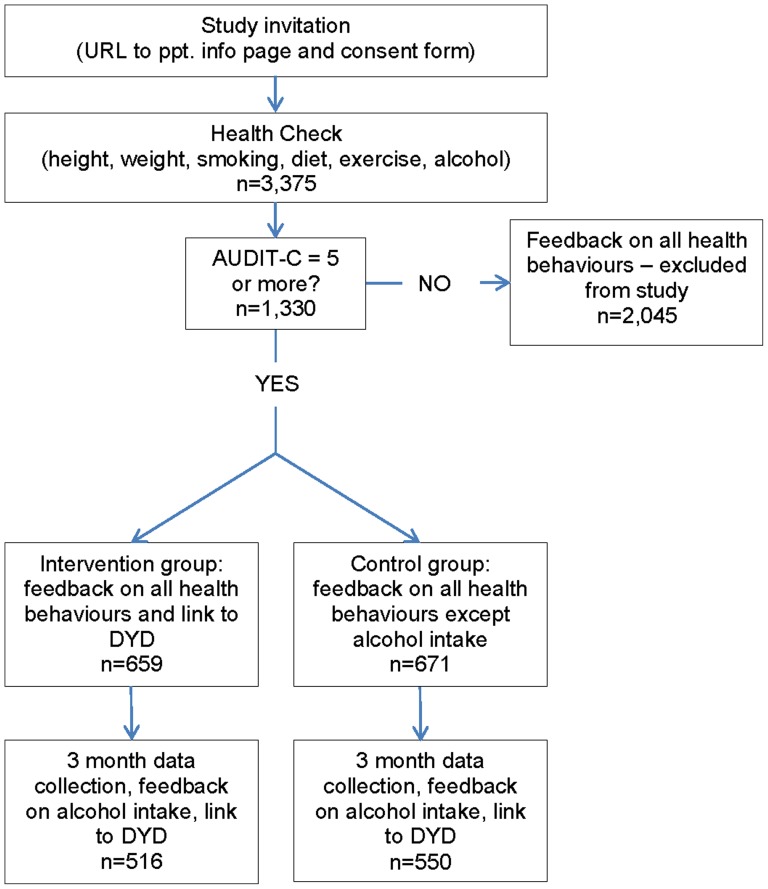
Consort diagram.

Of these, 1,330 (39%) scored five or more on the AUDIT-C and therefore entered the trial. Of those participants who were randomised to the intervention group and received feedback on their drinking (n = 659) (Figure 1) 19 (3%) registered with the Down Your Drink website for further help and support to reduce their drinking.

### Baseline characteristics

Participants were mostly male (75%), in their middle years (median age 48 years), married (77%), with children (68%). Half of participants were in managerial positions (55%). Around 12% of participants were smokers, median fruit and vegetable consumption was three portions a day and median time spent undertaking moderate and/or vigorous activity was 173 minutes a week. Participants in this study were slightly overweight (average BMI 26). All participants scored 5 or more on the AUDIT-C (indicating alcohol misuse) as this was necessary for inclusion in the trial. The median AUDIT-C score at baseline was six (Table 1).

**Table 1:** Demographics and health behaviours at baseline.

	Intervention Group	Control Group
	n = 659	n = 671
Sex Male, n (%)	503 (76.3%)	501 (74.7%)
Age years, median (IQR^a^)	48 (41 55)	48 (40, 53)
Ethnicity British, n (%)	597 (90.6%)	608 (90.6%)
Marital Status:		
Divorced, n (%)	65 (9.9%)	56 (8.4%)
Married, n (%)	509 (77.2%)	522 (77.8%)
Single, n (%)	85 (12.9%)	93 (13.9%)
Children, n (%)	452 (68.9%)	450 (67.4%)
Number of children, median (IQR)	2 (0, 2)	2 (0, 2)
Manager, n (%)	349 (53.0%)	376 (56.0%)
AUDIT-C^b^, median (IQR)	6 (5, 7)	6 (5, 8)
Current Smoker, n (%)	84 (12.8%)	81 (12.2%)
Body Mass Index kg/m^2^, median (IQR)	26.45 (24.21, 29.50)	26.18 (23.96, 28.82)
Portions of fruit and vegetables each day, median (IQR)	3 (2, 5)	3 (2, 5)
Exercise in last week:		
Vigorous mins, median (IQR)	60 (0, 150)	80 (0, 160)
Moderate mins, median (IQR)	105 (50, 210)	100 (60, 180)
Light mins, median (IQR)	17 (11, 26)	16 (10, 25)

a IQR  =  Interquartile range.

b Alcohol Use Disorders Identification Test – Consumption. Scores range from 0 to 12, where score of 5 or more indicates alcohol misuse [36], [37].

### Primary outcome

At three month follow-up, 1,066 (80%) participants completed follow-up questionnaires: 906 (68%) after email prompts, 71 (5%) after letter, 83 (6%) after telephone call, 6 (0.5%) unclear due to missing date data. Alcohol consumption (UK units consumed in the past week) was 5.6% higher in the intervention group who received instantaneous feedback on their drinking alongside feedback on other health behaviours compared with the wait-list control group who received no alcohol feedback, although this was not statistically significant (95% CI −4.7% to 16.9%; p = .30). The median number of units consumed a week in both intervention and control groups at follow-up was within recommended weekly limits (≤14 units per week for women; ≤21 units per week for men) women: intervention median 14 (Interquartile range 9 to 20) units a week, control median 12 (Interquartile range 7 to 17) units a week; men: intervention median 15 (Interquartile range 10 to 24) units a week, control median 15 (Interquartile range 9 to 23) units a week (Table 2).

**Table 2:** Outcomes by sex and randomised group at 3 months.

	Total				Male				Female			
	Control		Intervention		Control		Intervention		Control		Intervention	
	n = 671		n = 659		n = 337		n = 348		n = 138		n = 125	
Outcome measure	Median (IQR)	Mean (SD)	Median (IQR)	Mean (SD)	Median (IQR)	Mean (SD)	Median (IQR)	Mean (SD)	Median (IQR)	Mean (SD)	Median (IQR)	Mean (SD)
TOT-AL	16	19.06	17	20.25	15	16.62	15	18.35	12	13.81	14	14.75
(units^a^/week)	(10,25)	(14.36)	(11,26)	(15.10)	(9, 23)	(10.41)	(10,24)	(14.17)	(7, 17)	(9.53)	(9, 20)	(9.14)
AUDIT 10^b^	6	6.26	6	6.26	6	6.28	5	6.22	5	6.23	6	6.38
	(4, 7)	(2.79)	(4, 7)	(2.70)	(4, 8)	(2.67)	(4, 8)	(2.76)	(4, 7)	(3.11)	(4, 7)	(2.54)
AUDIT-C^c^	5	5.00	5	5.01	5	5.04	5	4.95	5	4.86	5	5.18
	(4, 6)	(1.31)	(4, 6)	(1.34)	(4, 6)	(1.34)	(4, 6)	(1.33)	(4, 6)	(1.22)	(4, 6)	(1.35)
Health utility^d^	1.00	0.89	1.00	0.89	1	0.89	1	0.89	1	0.9	1	0.90
	(0.8, 1)	(0.14)	(0.8, 1)	(0.16)	(0.8, 1)	(0.17)	(0.8, 1)	(0.14)	(0.9, 1)	(0.16)	(0.8, 1)	(0.15)

a 1 UK unit  = 8 grams ethanol.

b Alcohol Use Disorders Identification Test. Scores range from 0 to 40, where score of 8 or more indicates alcohol misuse [41].

c Alcohol Use Disorders Identification Test – Consumption. Scores range from 0 to 12, where score of 5 or more indicates alcohol misuse [36], [37].

d EQ-5D utility measure of quality of life [42].

### Secondary outcomes

There were no significant differences between experimental groups for the secondary outcomes. There was a difference of 0.01% (−4.3% to 4.5%; p = 1.0) in the AUDIT score. Health utility was −0.2% lower in the intervention group (−2.0% to 1.7%; p = 1.0).

### Post-hoc analysis

A post-hoc analysis was conducted to explore a possible floor effect by determining the difference in past week drinking between experimental groups among those participants scoring eight or more on the AUDIT-C at baseline. The analysis compared the 117 participants in the intervention group and 133 participants in the control group and found 14% higher alcohol consumption associated with the intervention (95% CI −5% to 30%; p = .15) however this was not statistically significant.

### Costing analysis

There were no significant differences in costs between groups (Table 3). The total mean cost, NHS plus employer cost, was lower in the intervention group, with a bootstrapped mean difference of £125 (95% CI −£52 to £302).

**Table 3:** Costs related to number of days of sickness absence and number and duration of hospital admissions in the past three months.

		Control	Intervention
Sick Leave	N	537	507
	Number with sick leave (%)	95 (17.7%)	80 (15.8%)
	Mean number of days if >0 (SD)	11 (20)	9 (16)
	Mean cost per person(SD)	293 (1510)	197 (1062)
NHS Costs			
GP appointments	Number	535	497
	Number with GP appointments (%)	235 (44.09%)	232 (46.68%)
	Mean number of appointments if >0 (SD)	1.7 (1.2)	1.8 (1.3)
	Mean cost per person(SD)	32 (50.8)	37 (56)
Outpatient appointments			
	Number with outpatient appointments (%)	62 (11.59%)	48 (9.66%)
	Mean number of appointments if >0 (SD)	2.2 (1.4)	2.5 (3)
	Mean cost per person(SD)	34.62 (117.34)	34.12 (169.32)
Day cases			
	Number with day case appointments (%)	17 (3.18%)	12 (2.41%)
	Mean number of appointments if >0 (SD)	1.6 (0.8)	2 (2.6)
	Mean cost per person(SD)	34.3 (211.46)	32.84 (332.85)
A&E attendances (admitted and not admitted)			
	Number with A&E attendances (%)	16 (2.99%)	21 (4.23%)
	Mean number of attendances if >0 (SD)	1.1 (0.25)	1.1 (0.30)
	Mean cost per person(SD)	2.6 (18.1)	3 (15.6)
Inpatient days (elective and emergency)			
	Number with inpatient admissions (%)	6 (1.12%)	3 (0.6%)
	Mean length of stay if admitted (SD)	2.2 (1.5)	2 (1.4)
	Mean cost per person(SD)	25.56 (264.68)	12.87 (202.42)
Total NHS cost	Mean cost per person(SD)	129.29 (437.91)	118.58 (451.08)
Total (sick leave plus mean cost)	Mean cost per person(SD)	422.41 (1663.29)	297.07 (1209.40)

## Discussion

### Main findings

The offer of an online health check attracted 3,375 (3%) employees from a large UK-based organisation in the three week recruitment period. Nearly 40% of employees who completed the health check scored five or more on the AUDIT-C (indicating alcohol misuse). At three month follow-up, there was no statistically significant difference in past week alcohol consumption between employees who did and did not receive online personalised feedback on their drinking in the context of a health check. There was also no difference between groups in the post-hoc analysis or in any of the secondary outcome measures, namely the AUDIT, EQ-5D index and costs. Nineteen (3%) participants in the intervention group accessed additional help to reduce their drinking via the Down Your Drink website.

### Comparison with existing literature

The findings of this trial do not support the existing literature which shows computer-based interventions may be effective in reducing alcohol consumption when compared with minimally active comparator groups, such as assessment-only or information-only websites [29]–[31]. However, the bulk of this emerging evidence base is conducted in student samples, with few studies conducted in the workplace. In one of the first studies to evaluate the effectiveness of an online personalised feedback intervention in the workplace setting (n = 218), participants were aged 18–24, were mostly female (73%), Single (75%) and attending school (75%) [45]. This study found that among high-risk participants (defined as meeting criteria for binge drinking), there were significantly greater reductions in weekend drinking and drinking to intoxication in participants receiving the intervention compared with those receiving the control (assessment-only), whereas no difference in drinking outcomes was found between experimental groups in low-risk participants [45]. The inclusion of non-drinkers or light drinkers was thought to dilute the intervention effect and offer a possible explanation for the neutral finding in a trial of online screening and personalised feedback on multiple risk behaviours (i.e. fruit and vegetable consumption, physical activity, smoking and binge drinking), based in a student health centre (n = 146) [46]. Participants in this study were aged 17–24, half were female (49%) and all were university students. At six week follow-up, there was no difference between groups (i.e. 1) intervention, 2) assessment-only and 3) minimal contact) in the proportion of students drinking within recommended limits for binge drinking. The inclusion of low-risk drinkers does not explain the neutral finding in our trial as all participants were drinking above recommended limits. There is no internationally agreed cut-off score for the AUDIT-C, with advocated thresholds for detecting hazardous drinking ranging from 2 to 5 in women and 3 to 6 in men [47]. The AUDIT-C cut-off score of 5 or more used in this trial reflects clinical guidance in England [37] and was most accurate in detecting drinking above UK weekly limits (>21 units/week for men and >14 units/week for women) in a trial of people seeking help with their drinking online (unpublished data) [48]. The neutral findings of this trial do not appear to be explained by a floor effect as our post-hoc analysis of participants with a baseline AUDIT-C score of eight or more found no benefits for the intervention.

A challenge that faces the interpretation of many trials of online interventions is that they evaluate access to the intervention, rather than engagement or use of it [48]-[50]. We do not know whether people read the feedback, particularly when it was presented alongside feedback on other health behaviours. Alternatively, the feedback may not have been perceived as relevant or valid, for example if the recommended limits are not seen as reliable. It may also be that the type of feedback was not effective at reducing alcohol intake, where effective online SBI in student populations often includes normative feedback [30], [51]. It is possible that the control condition may have been contaminated as the trial was conducted in one organisation although this would have required employees to share information about their responses to the questionnaire and the feedback obtained. It is also questionable whether personalised feedback delivered to someone else would impact on another person's drinking behaviour. This trial was supported by a small budget and conducted within a tight time-frame which militated against a qualitative exploration of the experience of people taking part in this study which may have illuminated the neutral findings. Future studies in this field would benefit from exploring the feasibility of delivering an online health check in the workplace, by considering the issues that affect participation and engagement with the intervention, along with the challenges of conducting a trial in this setting.

### Strengths and limitations

To increase the acceptability of the intervention and reduce selection bias, participants were invited to take part in a health check and receive personalised feedback as part of a study; they did not know they were taking part in a trial and that the focus of the study was on their alcohol consumption. The trial has low risk of bias from randomisation sequence generation, allocation to experimental groups and blinding of intervention facilitator and outcome assessor, as outlined in the Methods. The protocol was published to guard against reporting bias and the high rates of follow-up minimise the likelihood of response bias. This trial was designed to minimise the impact of assessment on alcohol consumption [52]–[55], with the 3-item AUDIT-C questionnaire the only alcohol-related measure used at baseline. Collection of AUDIT-C data was necessary to establish eligibility for the trial, therefore reactivity of assessment was minimised rather than eliminated and may have been responsible for the slight reduction in score within both groups at follow-up. Although this trial did not quite meet its pre-defined sample size, the 95% confidence intervals around the primary outcome were narrow and excluded the 20% reduction in alcohol intake used to power the sample size calculation. Therefore, the study was sufficiently powered and we can conclude with some certainty that there was no evidence of a difference between groups. The study team worked closely with the organisation's communications team to advertise the health check on the company's web-portal, in-line with their standard procedure; the trial could not have been conducted without the support and guidance of the occupational health lead.

Only 3% of the total workforce took part in the health check. Comparing the health behaviours of the participants in this study with the general adult population in England suggests that fewer of the participants were smokers (12% vs. 20% [56]), but median number of portions of fruit and vegetables was lower than average (3 portions/day vs. 4 portions/day [57]), as was median number of minutes of physical activity (173 minutes/week vs. 180 minutes/week [58]). In contrast, the proportion of employees exceeding the threshold for alcohol misuse (AUDIT-C score 5 or more) was higher than the national average (39% vs. 21% [58]). Whilst there was a large proportion of employees who exceeded the drinking threshold, the average score on the AUDIT-C was low (median 6). It is therefore unsurprising that there was no difference in alcohol-related harm when this population was unlikely to be experiencing problems at baseline. We do not know whether the health behaviours of the participants in this study are representative of this individual organisation. Long-term evaluation of between group differences was not possible in this trial due to the wait-list design and the imminent launch of the company's alcohol campaign, which would include a designated website with online tools for reducing drinking, opportunities for screening and feedback and possibly a road show. This campaign would have contaminated the study, therefore follow-up data was collected before the end of 2012 so that the company could launch their alcohol campaign in early January 2013.

## Conclusion

Online screening and personalised feedback on alcohol intake delivered in the context of a health check did not reduce alcohol intake among employees in a large UK-based private sector organisation when compared with online screening without alcohol feedback. The online health check attracted a relatively high proportion of employees who were drinking slightly above the threshold for alcohol misuse. Further research is needed to determine how to engage a larger proportion of employees to take part in screening.

## Supporting Information

Checklist S1 **CONSORT Checklist.**
(DOC)

Protocol S1 **Trial Protocol.**
(PDF)
