# Correction: Health on the Web: Randomised Controlled Trial of Online Screening and Brief Alcohol Intervention Delivered in a Workplace Setting

**DOI:** 10.1371/journal.pone.0127371

**Published:** 2015-04-27

**Authors:** Zarnie Khadjesari, Nick Freemantle, Stuart Linke, Rachael Hunter, Elizabeth Murray

In Table 1 and Table 2, there are errors in the data presented. Please see the corrected Table 1 and Table 2 here.

**Table 1:** Demographics and health behaviours at baseline.

	Intervention Group	Control Group
	n = 659	n = 671
Sex Male, n (%)	503 (76.3%)	501 (74.7%)
Age years, median (IQR^a^)	48 (41 55)	48 (40, 53)
Ethnicity British, n (%)	597 (90.6%)	608 (90.6%)
Marital Status:		
Divorced, n (%)	65 (9.9%)	56 (8.4%)
Married, n (%)	509 (77.2%)	522 (77.8%)
Single, n (%)	85 (12.9%)	93 (13.9%)
Children, n (%)	452 (68.9%)	450 (67.4%)
Number of children, median (IQR)	2 (0, 2)	2 (0, 2)
Manager, n (%)	349 (53.0%)	376 (56.0%)
AUDIT-C^b^, median (IQR)	6 (5, 7)	6 (5, 8)
Current Smoker, n (%)	84 (12.8%)	81 (12.2%)
Body Mass Index kg/m^2^, median (IQR)	26.45 (24.21, 29.50)	26.18 (23.96, 28.82)
Portions of fruit and vegetables each day, median (IQR)	3 (2, 5)	3 (2, 5)
Exercise in last week:		
Vigorous mins, median (IQR)	60 (0, 150)	80 (0, 160)
Moderate mins, median (IQR)	105 (50, 210)	100 (60, 180)
Light mins, median (IQR)	150 (60, 300)	140 (60, 280)

^a^IQR = Interquartile range

^b^Alcohol Use Disorders Identification Test—Consumption. Scores range from 0 to 12, where score of 5 or more indicates alcohol misuse [36, 37]

**Table 2:** Outcomes by sex and randomised group at 3 months.

	Total				Male				Female			
	Control		Intervention		Control		Intervention		Control		Intervention	
	n = 671		n = 659		n = 501		n = 503		n = 170		n = 156	
Outcome measure	Median (IQR)	Mean (SD)	Median (IQR)	Mean (SD)	Median (IQR)	Mean (SD)	Median (IQR)	Mean (SD)	Median (IQR)	Mean (SD)	Median (IQR)	Mean (SD)
TOT-AL	16	19.06	17	20.25	18	20.0	18	21.47	12	16.11	15	16.18
(units^a^/week)	(10,25)	(14.36)	(11,26)	(15.10)	(10,27)	(14.27)	(11,27)	(16.13)	(9, 18)	(14.29)	(9, 23)	(10.01)
AUDIT 10^b^	6	7.23	6	7.14	6	7.40	6	7.12	6	6.72	6	7.21
	(5, 9)	(3.87)	(5, 9)	(3.65)	(5, 9)	(3.95)	(5, 9)	(3.61)	(4, 8)	(3.59)	(5, 8.5)	(3.81)
AUDIT-C^c^	5	5.44	5	5.41	5	5.54	5	5.40	5	5.14	5	5.46
	(4, 6)	(1.59)	(4, 6)	(1.63)	(4, 6.5)	(1.61)	(4, 6)	(1.68)	(4, 6)	(1.51)	(4, 6)	(1.49)
Health utility^d^	1.00	.89	1.00	.89	1	.89	1	.89	1	.90	1	.90
	(.80, 1)	(.16)	(.80, 1)	(.14)	(.80, 1)	(.17)	(.80, 1)	(.14)	(.85, 1)	(.15)	(.80, 1)	(.14)

^a^1 UK unit = 8 grams ethanol

^b^Alcohol Use Disorders Identification Test. Scores range from 0 to 40, where score of 8 or more indicates alcohol misuse [41]

^c^Alcohol Use Disorders Identification Test—Consumption. Scores range from 0 to 12, where score of 5 or more indicates alcohol misuse [36, 37]

^d^EQ-5D utility measure of quality of life [42]

There is an error in the last sentence of the Primary outcome subsection of the Results. The correct sentence is: The median number of units consumed a week in both intervention and control groups at follow-up was within recommended weekly limits (≤14 units per week for women; ≤21 units per week for men), with the exception of women in the intervention group (Table 2).
